# Enhancement in hydrogen evolution using Au-TiO_2_ hollow spheres with microbial devices modified with conjugated oligoelectrolytes

**DOI:** 10.1038/npjbiofilms.2015.20

**Published:** 2015-10-21

**Authors:** Chee Keong Ngaw, Victor Bochuan Wang, Zhengyi Liu, Yi Zhou, Staffan Kjelleberg, Qichun Zhang, Timothy Thatt Yang Tan, Say Chye Joachim Loo

**Affiliations:** 1 Energy Research Institute @ NTU (ERI@N), Interdisciplinary Graduate School, Nanyang Technological University, Singapore, Singapore; 2 Solar Fuels Laboratory, School of Chemical and Biomedical Engineering, Nanyang Technological University, Singapore, Singapore; 3 Solar Fuels Laboratory, School of Materials Science and Engineering, Nanyang Technological University, Singapore, Singapore; 4 School of Materials Science and Engineering, Nanyang Technological University, Singapore, Singapore; 5 Singapore Centre on Environmental Life Sciences Engineering (SCELSE), Nanyang Technological University, Singapore, Singapore; 6 School of Biotechnology and Biomolecular Sciences and Centre for Marine Bio-Innovation, The University of New South Wales, Sydney, New South Wales, Australia

## Abstract

**Objective:**

Although photoelectrochemical (PEC) water splitting heralds the emergence of the hydrogen economy, the need for external bias and low efficiency stymies the widespread application of this technology. By coupling water splitting (in a PEC cell) to a microbial fuel cell (MFC) using *Escherichia coli* as the biocatalyst, this work aims to successfully demonstrate a sustainable hybrid PEC–MFC platform functioning solely by biocatalysis and solar energy, at zero bias. Through further chemical modification of the photo-anode (in the PEC cell) and biofilm (in the MFC), the performance of the hybrid system is expected to improve in terms of the photocurrent generated and hydrogen evolved.

**Methods:**

The hybrid system constitutes the interconnected PEC cell with the MFC. Both PEC cell and MFC are typical two-chambered systems housing the anode and cathode. Au-TiO_2_ hollow spheres and conjugated oligoelectrolytes were synthesised chemically and introduced to the PEC cell and MFC, respectively. Hydrogen evolution measurements were performed in triplicates.

**Results:**

The hybrid PEC–MFC platform generated a photocurrent density of 0.35 mA/cm^2^ (~70× enhancement) as compared with the stand-alone P25 standard PEC cell (0.005 mA/cm^2^) under one-sun illumination (100 mW/cm^2^) at zero bias (0 V vs. Pt). This increase in photocurrent density was accompanied by continuous H_2_ production. No H_2_ was observed in the P25 standard PEC cell whereas H_2_ evolution rate was ~3.4 μmol/h in the hybrid system. The remarkable performance is attributed to the chemical modification of *E. coli* through the incorporation of novel conjugated oligoelectrolytes in the MFC as well as the lower recombination rate and higher photoabsorption capabilities in the Au-TiO_2_ hollow spheres electrode.

**Conclusions:**

The combined strategy of photo-anode modification in PEC cells and chemically modified MFCs shows great promise for future exploitation of such synergistic effects between MFCs and semiconductor-based PEC water splitting.

## Introduction

Achieving a hydrogen economy can alleviate the universal fossil fuel crunch and rampant pollution driven by the insatiable demand for energy. Solar-illuminated semiconductor-based photoelectrochemical (PEC) water splitting is an attractive strategy to generate hydrogen, which has witnessed significant breakthroughs recently.^1–4^ Titanium dioxide (TiO_2_) and iron oxide (Fe_2_O_3_) have been extensively studied as common materials for application as photo-anodes in PEC cells owing to their favourable optoelectronic properties.^1,4^ However, there is much to improve on, such as enhancing visible light absorption capabilities to maximise the full solar spectrum.^5–7^ In particular, TiO_2_ and Fe_2_O_3_ can only absorb in certain regions of the solar spectrum. This can be addressed through the synthesis of novel nano-structured hybrid materials, which can be tailored to manipulate material composition, shape, size and geometrical configurations.^8^ To fully exploit the complete solar spectrum, gold nanoparticles (AuNPs) can also be used to functionalize TiO_2_ to impart strong visible light photoabsorption capabilities, specifically at ~550 nm.^5^ This phenomenon can be attributed to localised surface plasmon resonance, which is characteristic of AuNPs.^9,10^ Further, hollow spheres morphology can be fabricated to increase the surface area for photocatalytic reactions. In addition, AuNPs entrapped within the hollow sphere architecture can facilitate direct charge transfer from metal to TiO_2_ and function as traps to minimise electron and energy back flow in the form of surface charge recombination.^11,12^

Although PEC water splitting may hold the key to achieving a hydrogen economy, this phenomenon needs to be driven by an external bias, which decreases the competitiveness of this platform. To demonstrate a self-sustaining water splitting device driven by solar energy, a continual supply of external energy must be provided.

Microbial fuel cells (MFCs) are a form of bioelectrochemical system that relies on the inherent charge transfer mechanism(s) possessed by the bacterial strain to generate bioelectricity.^13,14^ Biocatalysis of organic compounds releases electrons and protons at the anaerobic anode, which is coupled to oxygen reduction at a cathode. Although the electrical output of this platform remains limited, much remains to be explored from the microbial aspect. A thorough understanding of microbial charge transfer capabilities is needed for maximum exploitation.^15^ Recent advances have included chemical modification of the bacterial membrane through spontaneous intercalation of synthetic molecular analogues to improve device performance.^16,17^ This class of conjugated oligoelectrolytes (COEs), specifically, 4,4'-bis(4'-(*N*,*N*-bis(6''-(*N*,*N*,*N*-trimethylammonium)hexyl)amino)-styryl)stilbene tetraiodide (DSSN+), are built from electronically delocalised π-conjugated aromatic backbones with pendant ionic groups, which confers solubility in aqueous media. Its amphiphilic nature allows for insertion and self-alignment within lipid membranes. Although the exact charge transfer mechanism afforded by this approach still remains debatable, it is evident that this strategy can improve charge collection in *Escherichia coli-* and wastewater-based MFCs.^16,18,19^

In a hybrid system comprising MFC and PEC cells, the combined platform has the potential to realise a solar-microbial device that can produce hydrogen (from PECs) through electrons liberated by microbial oxidation of organic substrates (in MFCs), at zero bias. In this case, if ample sunlight and a continuous flow of organic compounds are supplied, the hybrid system is expected to be self-sustaining in nature. Although this hybrid technology is relatively new, it has already been used in wastewater systems.^20^ In this contribution, a double-pronged approach is used to demonstrate enhanced hydrogen evolution through an integrated PEC–MFC platform—(1) using Au-TiO_2_ hollow spheres to improve the photoabsorption capabilities of the photo-anode through enhanced surface plasmon resonance effect in PEC cells and (2) chemical functionalization of *E. coli* with two novel conjugated oligoelectrolytes, i.e., DSSN+ and 6,6',6'',6'''-(((-thiophene-2,5-diylbis(ethene-2,1-diyl))bis(4,1 phenylene))bis(azanetriyl))tetrakis(N,N,N-trimethylhexan-1-aminium) iodide (DSTN+), to enhance extracellular charge transfer in MFCs. The performance of the hybrid system is investigated by linear sweep voltammograms (LSVs) and chopped amperometric current–time curves. The amount of evolved H_2_ is further quantified and shown to be greatly enhanced in the modified hybrid system. This successful demonstration points towards the possibility of using various strategies to enhance PEC–MFC platforms for enhanced hydrogen evolution.

## Materials and Methods

### Synthesis of Au-TiO_2_ hollow spheres

Fabrication of Au-TiO_2_ hollow spheres was performed according to literature.^5^ Briefly, Au-carbon nanospheres were synthesised through the emulsion polymerisation reaction of HAuCl_4_/glucose solution under hydrothermal conditions. Newly formed Au-carbon nanospheres were dispersed in a titanium isopropoxide solution (20 ml, 3 mol/l) through ultra-sonication for 10 min, to ensure that the carbon nanospheres were fully dispersed before Ti precursor was added to it. A 500 W ultra-sonicator (50 Hz) was used. The Ti-carbon nanospheres solution was aged for 18 h at room temperature and further filtered, washed and dried at 80 °C for 12 h. The resultant composite nanospheres were then placed in a furnace and heated to 550 °C in air at a heating rate of 16 °C/min, further held at 550 °C for 1 h and cooled naturally to room temperature. TiO_2_ hollow spheres were synthesised with similar procedures.

### Doctor blade technique

The doctor blade technique is a screen-printing method commonly used for the fabrication of photo-electrodes. This technique consists of two main components- (1) the process of converting the nanocomposites (Au-TiO_2_) into a smooth paste and applying it onto the surface of the fluorine doped tin oxide (FTO) glass to make it into a photo-electrode and (2) the as-prepared photo-electrode will have to be calcinated at high temperatures for the removal of any impurities present on the electrode surface. It is important to note that the preparation of the Au-TiO_2_ paste as well as the calcination step would have a significant influence on the quality of the surface of the photo-electrode, therefore optimisation of these two components will be crucial for the fabrication of a good photo-electrode with high photocurrent output.

### Preparation of Au-TiO_2_ paste

6 g of the as-prepared Au-TiO_2_ hollow spheres were transferred into an alumina mortar in powder form. Acetic acid (1 ml), water (1 ml) and ethanol (1 ml) were added to improve the stability of the paste from cracking. At each addition, the specific solvent was introduced dropwise and the paste was further ground into fine particles using a mortar. After grinding for 15 min, 20 g of terpineol and 3 g of ethyl cellulose (10% solution in ethanol) were added to the resultant powder, which was further ground into a smooth paste.^21^

### Preparation of Au-TiO_2_ electrode

The FTO glass (15 Ω, 25×11×2.2 mm) was used as a current collector. Thorough cleaning was performed by subjecting the FTO glass to three cleaning cycles consisting of ultra-sonication in water, acetone and ethanol. The as-prepared Au-TiO_2_ paste was spread evenly onto the surface of the FTO glass using the doctor blade technique.^21–23^ The electrode was dried for 5 min at room temperature and gradually heated in the muffle furnace at various conditions (325 °C/5 min, 375 °C/5 min, 450 °C/15 min, 500 °C/15 min) through a heating cycle (Supplementary Figure S1, Supplementary Information) and subsequently left to cool to room temperature naturally to obtain the final product. The TiO_2_ hollow spheres and P25 electrodes were synthesised with similar procedures.

### Characterisation of Au-TiO_2_ hollow spheres

The morphology of the Au-TiO_2_ hollow spheres was characterized by both field emission scanning electron microscopy (JEOL JSM-7600F) and transmission electron microscopy (JEOL 2100) at an acceleration voltage of 200 kV. X-ray power diffraction (XRD) patterns were recorded using a (Shimadzu, Singapore, Singapore) XRD-6000 X-ray diffractometer (Cu-Kα source) at a scan rate of 1°/min with 2*θ* ranging from 20 to 80°. Ultraviolet–visible absorption spectra of the Au-TiO_2_, TiO_2_ and P25 films were obtained using a Lambda 750 UV/Vis/NIR spectrophotometer (Perkin Elmer, Singapore, Singapore) with BaSO_4_ as a reference.

### Synthesis of DSSN+ and DSTN+

The synthesis of DSSN+ was performed according to literature.^24^ DSTN+ synthesis procedures and characterisation are listed in Supplementary Information.

### PEC cell assembly

The PEC cell setup consists of a two-electrode configuration system. A platinum wire was used as the counter and reference electrode. A Nafion proton exchange membrane (2 cm in diameter) was placed between the anode and cathode compartments to facilitate the movement of protons within the cell. The working surface area of the PEC cell was ~3.14 cm^2^.

### MFC assembly

All the materials were used as received, unless otherwise stated. U-tube dual-chamber MFCs were assembled according to literature^15,16,25^ (Supplementary Figure S2, Supplementary Information). Two 90° ball-to-plain-end and socket-to-plain-end glass tubes (17 mm O.D.×1.8 mm wall thickness; Spectra-Teknik, Singapore, Singapore) were joined using a stainless steel pinch clamp (#28; Spectra-Teknik) and high vacuum silicone grease. The interface between both tubes was formed by a circular (diameter of 2 cm) piece of Nafion N117 proton exchange membrane (Ion Power, New Castle, DE, USA). The dimensions of carbon felt electrodes (3.18 mm thickness; VWR Singapore Pte. Ltd., Singapore, Singapore) were 5 cm×2 cm (length×width). Titanium wire was connected to the electrodes by plastic screws and nuts (Spectra-Teknik). The anode chamber was covered with a silicone septum and the titanium wire was threaded through, whereas the cathode chamber was covered loosely with an inverted glass vial to provide an aerobic environment. In addition, the cathode electrode was only partly submerged to allow oxygen reduction at the aerobic section of the device architecture. The glass tubes were then filled with ultrapure water and autoclaved for sterility. After sterilising and decanting the ultrapure water, the anode chamber was filled with 10 ml of lysogeny broth and 10 ml of bacterial culture. The cathode contained 20 ml of lysogeny broth. The electrodes were then connected to 1 kΩ resistors and the voltage was recorded with an eDAQ e-corder data acquisition system (Bronjo Medi, Singapore, Singapore) at a rate of one point per 5 min. *E. coli* was cultured overnight in lysogeny broth at 37 °C and adjusted to OD_600_ ~1.0 before inoculation into the MFC anode only.

### Interconnection in the PEC–MFC hybrid system

The independent platforms are interconnected via wires to form a hybrid system. The MFC anode is connected to the counter electrode of the PEC cell, whereas the photo-anode of the PEC cell is connected to the MFC cathode (Supplementary Figure S2, Supplementary Information). Robust interconnects are confirmed through probing by a multimeter.

### Electrical characterisation of devices

LSVs of stand-alone PEC cells and PEC–MFC hybrid systems were measured in a two-electrode configuration with platinum wire as the counter and reference electrode. For the PEC–MFC hybrid system, the Au-TiO_2_ hollow spheres, TiO_2_ hollow spheres and standard P25 electrodes functioned as the working electrode. All electrical characterisation was performed using a CHI 660D electrochemical work station (CH Instruments, Singapore, Singapore) at a scan rate of 20 mV/s. The electrolyte was 0.5 M Na_2_SO_4_ solution (pH 7.0). The effective surface area of the working electrode was 0.2 cm^2^. The light source was simulated from a 150 W xenon solar simulator (96000, Newport Corporation, Singapore, Singapore) using a solar filter with a measured intensity equivalent to standard AM1.5 sunlight (100 mW/cm^2^).

### Quantification of hydrogen evolution

The H_2_ produced at the platinum electrode in the modified PEC–MFC hybrid system was collected using a syringe after 36 h and analysed with a gas chromatograph (Agilent 7890A, Singapore, Singapore). The electrolyte was degassed by purging N_2_ gas for 30 min to ensure absence of residual gas contaminants. The H_2_ measurements were carried out manually by using a syringe to extract the evolved H_2_ from the void space of the PEC cell and subsequently injected into the gas chromatograph at an interval of 1 h. All the measurements were repeated three times to ensure that the results obtained were accurate (where *n*=3).

## Results

**Figure 1:**
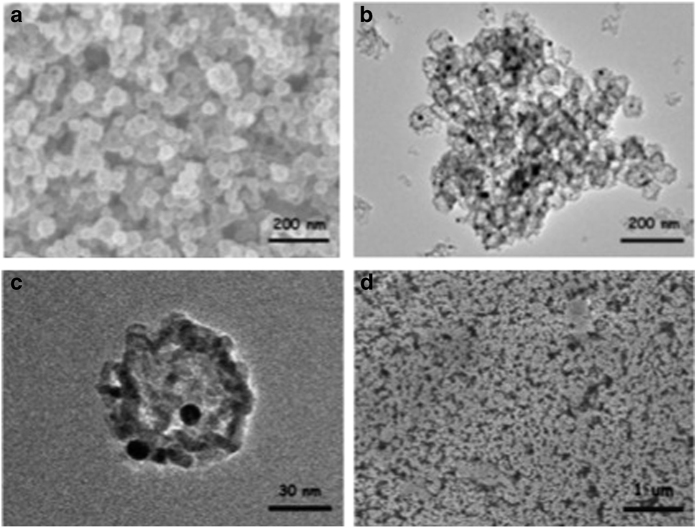
Topological characterisation of Au-TiO_2_ hollow spheres. (**a**) FESEM image of Au-TiO_2_ hollow spheres. (**b**) TEM image showing formation of hollow spheres. (**c**) Enlarged TEM image showing AuNP encased within hollow sphere. (**d**) FESEM of electrode consisting Au-TiO_2_ hollow spheres. AuNP, gold nanoparticle; FESEM, field emission scanning electron microscopy; TEM, transmission electron microscopy.

The Au-TiO_2_ hollow spheres were used as the working electrode in the PEC cell owing to its favourable band position for PEC water splitting, good chemical stability and low cost.^26–28^ In addition, AuNPs embedded within the TiO_2_ hollow spheres can improve charge separation within the electrode by reducing recombination reactions. The Au-TiO_2_ hollow spheres were prepared via a carbonaceous hard template strategy.^5^ The field emission scanning electron microscopy image shows that the Au-TiO_2_ hollow spheres have an average size of ~50–70 nm (Figures 1a and b). Transmission electron microscopy images revealed the formation of hollow spheres with an average diameter of ~60 nm and the presence of AuNPs encased within the internal cavity of the hollow spheres (Figure 1c). The Au-TiO_2_ hollow spheres were then processed into a smooth paste and deposited onto the surface of an FTO glass substrate by the doctor blade technique.^21^ The field emission scanning electron microscopy image collected from the thin film on the FTO surface reveals a densely packed array of nanoparticles with an average diameter of ~60 nm (Figure 1d).

**Figure 2:**
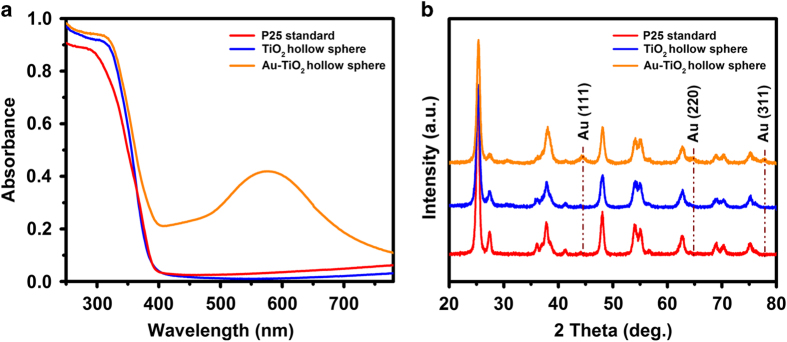
(**a**) Optical absorption spectroscopy and (**b**) X-ray diffraction patterns of various electrodes used in PEC cells. PEC, photoelectrochemical.

Optical absorption spectroscopy was performed to further analyse the fabricated electrodes (Figure 2a). All the electrodes exhibited an intensive absorption band at wavelengths shorter than 390 nm, which is consistent with the intrinsic band gap reported for TiO_2_ (~3.2 eV).^29^ However, in contrast to the TiO_2_ hollow spheres and P25 standard electrodes, the Au-TiO_2_ hollow spheres electrode exhibited a characteristic plasmonic absorption band at ~580 nm. This is attributed to the surface plasmon resonance effect of the AuNPs, whereas the TiO_2_ hollow spheres and P25 standard electrodes do not exhibit such peaks. In addition, XRD spectra from the respective electrodes were collected (Figure 2b). TiO_2_ hollow spheres and P25 standard films showed similar XRD profiles and exhibited strong diffraction peaks at 25.3°, 37.9°, 48.1°, 54.0° and 62.7°, which correspond to the anatase faces of (101), (004), (200), (105) and (204) of TiO_2_, respectively (JPCDS No. 21.1272). Minor diffraction peaks were also detected at 27.4° and 41.2°, which correspond to the rutile faces of (110) and (200) of TiO_2_, respectively (JCPDS No. 76–1940). Collectively, these results reveal that the crystal structures of TiO_2_ hollow spheres and P25 standard films exhibit a mixture of anatase and rutile phases. In contrast to the XRD patterns of TiO_2_ hollow spheres and P25 standard films, the XRD pattern of Au-TiO_2_ hollow spheres exhibited additional diffraction peaks at 44.5°, 68.4° and 77.5°, which can be attributed to the (111), (220) and (311) faces of Au (JCPDS No. 65–8601). This analysis indicates the successful incorporation of AuNPs within the TiO_2_ hollow spheres.

**Figure 3:**
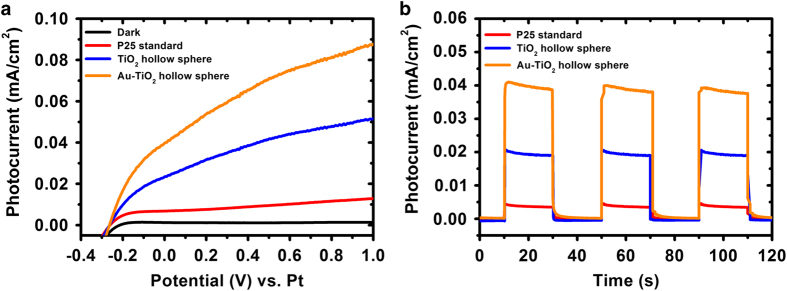
(**a**) Linear sweep voltammograms and (**b**) amperometric current–time curves of stand-alone PEC cell using different photo-anodes. PEC, photoelectrochemical.

Electrical performances of individual PEC cells were analysed to investigate the role of photo-anode modification in PEC water splitting. LSVs of the various photo-anodes were performed in 0.5 M Na_2_SO_4_ electrolyte under one-sun illumination (AM1.5, 100 mW/cm^2^; Figure 3a). At 0 V, the Au-TiO_2_ hollow spheres produced the highest photocurrents (~0.04 mA/cm^2^, orange trace), whereas TiO_2_ hollow spheres produced ~0.02 mA/cm^2^ (blue trace). Last, the system using standard P25 produced ~0.005 mA/cm^2^ (red trace). LSV under dark condition (black trace) exhibited a horizontal line and negligible current density was generated at 0 V, indicating that current does not increase significantly when swept under dark conditions. Collectively, these results validate the choice of Au-TiO_2_ hollow spheres as the best material, among these candidates, for the photo-anode. Amperometric on–off current–time curves further corroborate these observations (Figure 3b).

The observed photocurrent for P25 standard increased from ~0.005 mA/cm^2^ to ~0.02 mA/cm^2^ for TiO_2_ hollow spheres. The enhanced photocurrent generation (~4×) is attributed to the hollow sphere morphology as it is a favourable architecture for photocatalytic activities.^9,30,31^ Photons penetrating through the shell reflect from the walls of the sphere to induce sustainable reflection within the hollow cavity. This enhances the probability of light absorption by the TiO_2_ for better efficiency. In addition, it is interesting to observe that incorporating AuNPs into the TiO_2_ hollow spheres can further improve the photocurrent generation to ~0.04 mA/cm^2^. This enhanced performance is attributed to the presence of AuNPs encased within the cavity of the hollow spheres. The AuNPs not only induce surface plasmon resonance effect, which generates more photoelectrons for higher photocurrent output,^32–34^ they can also function as electron sinks^35,36^ to ensure reduced charge recombination within the Au-TiO_2_ architecture for enhanced photocatalytic effect. No hydrogen gas was produced at the Pt wire for the stand-alone PEC cell, which is owing to the low photocurrent output produced at zero bias (0 V vs. Pt).

**Figure 4:**
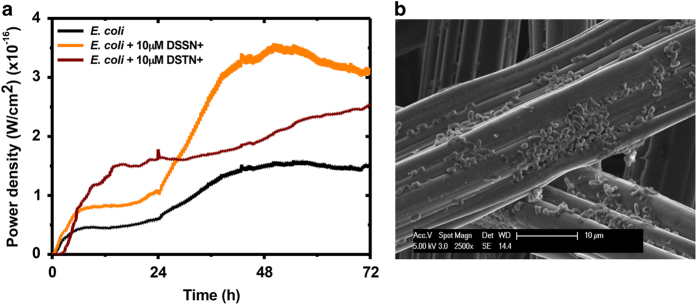
(**a**) Power density vs. time of MFC inoculated with *E. coli* chemically modified with DSSN+ and DSTN+. Geometrical area of electrode is 20 cm^2^. Data represent the average of triplicates. (**b**) Scanning electron microscopy image of *E. coli* biofilm colonized on a carbon fibre electrode surface. MFC, microbial fuel cell.

Currently, two hypotheses exist to account for the enhanced extracellular charge transfer afforded to COE-modified bacterial systems—(1) direct charge transfer across the conjugated molecular backbone of the intercalated COEs, which are functioning as nanowires within the cellular membrane;^18,24^ (2) membrane perturbation after COE intercalation in the cellular membrane, causing concomitant leakage of intracellular redox active component, which contributes to extracellular charge transfer.^17,37,38^ Although the specific mechanism(s) remain in contention, MFCs using such chemically modified *E. coli* shows enhanced electrical output. The independent MFCs using *E. coli* as the biocatalyst without chemical modification exhibited a maximum power density of ~1.5×10^−16^ W/cm^2^. The addition of 10 μM DSTN+ and DSSN+ improved the electrical output to ~2.5×10^−16^ W/cm^2^ and ~3.5×10^−16^ W/cm^2^, respectively (Figure 4a). In an attempt to improve charge transfer by using such COEs, the chemical backbone of DSTN+ has been modified by replacing one of the benzene ring, as seen in DSSN+, with a thiophene moiety, which is hypothesised to have better charge transfer capabilities.

Scanning electron microscopy image clearly revealed biofilm colonizing on the electrode surface, which has been retrieved from the anode chamber of the MFC (Figure 4b). The cells are rod shaped (width of ~0.5 μm and length of ~2 μm), which are typical morphologies of *E. coli*. Upon COE incorporation into the cellular membrane of *E. coli*, enhanced charge transfer will occur via one or both of the aforementioned hypotheses. Either of the proposed mechanisms can be the major route for enhanced charge facilitation, however, this specific investigation does not fall within the scope of this study, and is independent of the conclusions in this work.

**Figure 5:**
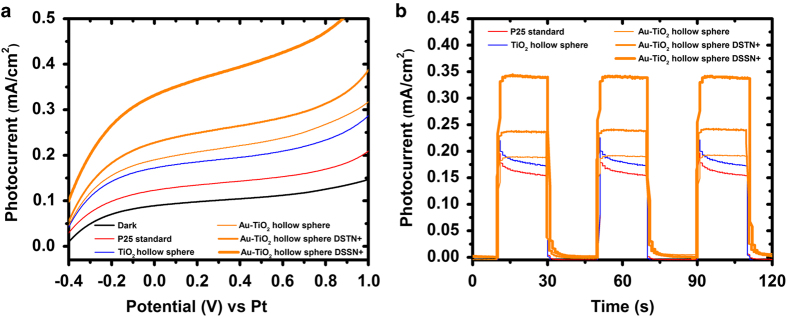
(**a**) Linear sweep voltammograms and (**b**) amperometric current–time curves of hybrid PEC–MFC system using different photo-anodes and *E. coli* chemically modified with DSTN+ and DSSN+. The electrolyte is 0.5 M Na_2_SO_4_ and the scan rate is 20 mV/s. Poising potential is 0 V vs. Pt, with light on–off cycle at light intensity of 100 mW/cm^2^. MFC, microbial fuel cell; PEC, photoelectrochemical.

The combined effects of COE-incorporated *E. coli* and modified photo-anodes were investigated in the PEC–MFC hybrid system. To gain deeper insight, three different types of photo-anodes (Au-TiO_2_ hollow spheres, TiO_2_ hollow spheres and P25 standard) were fabricated as PEC cells. MFCs using chemically modified *E. coli* were interfaced to the different PEC cells in series by connecting the MFC anode electrode to the Pt cathode in the PEC cell and subsequently, the MFC cathode electrode to the photo-anode in the PEC cell. As the MFCs will only reach its maximum power density after 36 h (Figure 4a), all electrical measurements performed on the PEC–MFC hybrid system will commence after 36 h from the point the MFC was inoculated with *E. coli*. LSV data were collected from the PEC–MFC hybrid systems modified with different COEs and photo-anodes in the dark and under one-sun illumination (Figure 5a). It is interesting to note that the LSV for the PEC–MFC hybrid system in dark condition (black trace, Figure 5a) generated a photocurrent of ~0.08 mA/cm^2^ at zero bias (0 V vs. Pt). This value is substantially larger than that for individual PEC cells (black trace, Figure 3a) at the same potential. This enhancement is attributed to the liberated electrons originating from the MFC in the hybrid system. *E. coli* oxidises organic matter in the anode media and releases electrons which will flow through the external circuit to the Pt wire of the PEC cell, hence producing current in the process. This demonstration validates the hypothesis that integrating an MFC to a PEC cell can enhance the performance of the resultant hybrid system, which shows promise in liberating hydrogen gas at zero bias (0 V vs. Pt).

The P25 standard photo-anode used in the PEC–MFC hybrid system without COE modification exhibited a photocurrent of ~0.12 mA/cm^2^ at zero bias (0 V vs. Pt; Figure 5a). This enhancement is ~30× larger than the photocurrent obtained for the stand-alone PEC cell using the same photo-anode (~0.005 mA/cm^2^, red trace in Figure 3a) at the same potential. This further confirms the validity of the adopted PEC–MFC hybrid platform. It is noteworthy to mention that the photocurrent of the PEC–MFC hybrid system using P25 standard photo-anode increases from ~0.12 mA/cm^2^ to ~0.16 mA/cm^2^ and 0.18 mA/cm^2^ after changing the photo-anode to TiO_2_ hollow spheres and Au-TiO_2_ hollow spheres respectively. This observed trend in increased photocurrent is in line with changes in the hollow spheres architecture as well as the reduced recombination mechanism afforded by using AuNPs, as reported in literature.^33,39^

In comparison with the PEC–MFC hybrid system using unmodified *E. coli*, higher photocurrents were observed across all three photo-anodes in systems using chemically modified *E. coli* at zero bias (Figure 5a and Supplementary Figure S3a). This illustrates the effect of such COEs in significantly enhancing the photocurrent generation in the modified hybrid systems through improved performance in the modified MFCs. The hybrid system using Au-TiO_2_ hollow spheres in the PEC cell and DSSN+ in the MFC generated the highest photocurrent (~0.35 mA/cm^2^) through an additive effect at zero bias (0 V vs. Pt). This enhancement is ~9× larger than the photocurrent obtained for the stand-alone PEC cell using only Au-TiO_2_ hollow spheres (~0.04 mA/cm^2^, orange trace in Figure 3a) at the same potential. It is worth mentioning that a lower photocurrent (~0.23 mA/cm^2^) was observed for the PEC–MFC hybrid system using Au-TiO_2_ hollow spheres/DSTN+ as compared with Au-TiO_2_ hollow spheres/DSSN+ (~0.35 mA/cm^2^). The inferior performance of DSTN+ as compared with DSSN+ is unknown at this point and is subject to future investigations. However, it should be pointed out that COE modification of *E. coli* resulted in enhanced performances in the hybrid systems. Amperometric on–off current–time curves corroborate these observations (Figure 5b and Supplementary Figure S3b). These data further confirm earlier observations that COE-modified *E. coli* MFCs produce higher power density, especially so in the DSSN+-modified bacterial system, and Au-TiO_2_ hollow spheres absorb the most photons as compared with systems using TiO_2_ hollow spheres and P25 standard photo-anode. In addition, the stability of the PEC–MFC hybrid system using Au-TiO_2_ hollow spheres and DSSN+ was investigated by measuring the photocurrent of the system over time under one-sun illumination (100 mW/cm^2^). It was found that the hybrid system demonstrated reproducible photocurrent values without any significant drop within 4000 s. This result clearly shows that the adopted system and various modifications are stable and efficient for continuous operation without any zero bias (0 V vs. Pt).

## Discussion

**Figure 6:**
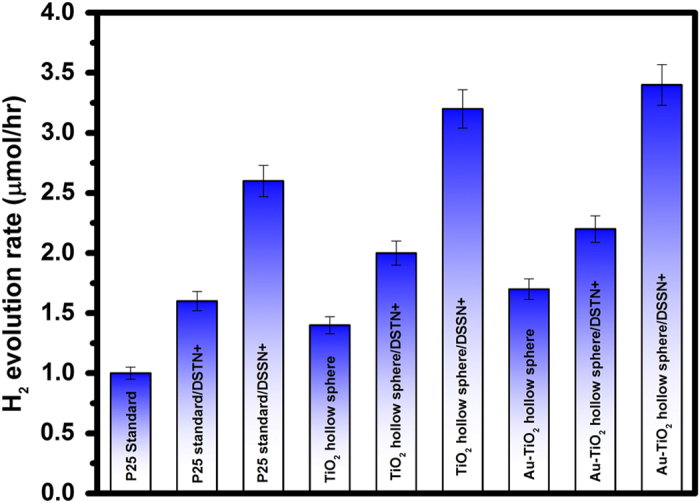
Hydrogen evolution profile of the hybrid PEC–MFC system after 36 h with chemically modified photo-anodes and COE-incorporated *E. coli*. COE, conjugated oligoelectrolyte; MFC, microbial fuel cell; PEC, photoelectrochemical.

Hydrogen concentration was quantified in the modified PEC–MFC hybrid system after 36 h to elucidate the effects of the modifications on hydrogen evolution (Figure 6). The hydrogen evolution rate of the PEC–MFC hybrid system using Au-TiO_2_ hollow spheres photo-anode and DSSN+-incorporated *E. coli* was the highest (~3.4 μmol/h) at zero bias. Consequently, the system using only P25 standard electrode (without any modification) exhibited a hydrogen evolution rate of ~1.0 μmol/h at the same potential. This result strongly suggests that the amount of H_2_ produced is highly dependent on the photocurrent generated by the PEC–MFC hybrid system. In addition, the solar-to-hydrogen efficiencies (STH) of the PEC–MFC hybrid systems using Au-TiO_2_ hollow spheres photo-anode/DSSN+-incorporated *E. coli* and P25 standard electrode were calculated using the following equation.^40^

$$\mathrm{STH}=\frac{j\times (1.23–V)}{P_{\mathrm{sun}}}$$

where V is the potential difference between the photo-anode and Pt cathode, *j* (mA/cm^2^) is the photocurrent density at the specified voltage, *P*_sun_ is the irradiance intensity at 100 mW/cm^2^ (AM1.5 sunlight). The P25 standard electrode in the hybrid system produced an efficiency of 0.19% at zero bias (0 V vs. Pt), while the PEC–MFC hybrid system using Au-TiO_2_ hollow spheres photo-anode/DSSN+-incorporated *E. coli* achieved an improved efficiency of 0.44% at the same potential. Faradaic efficiencies for the hybrid systems (Au-TiO_2_ hollow spheres photo-anode/DSSN+-incorporated *E. coli* and P25 standard electrode) producing hydrogen gas were also calculated to be 78.9 and 75.4%, respectively. This observation suggests that the amount of H_2_ evolved was less than expected and can be attributed to the charge recombination occurring at the Au-TiO_2_ hollow spheres photo-anode. When the electrode was illuminated, ‘hot’ plasmonic electrons from AuNPs will be injected into the conduction band of TiO_2_, while ‘hot’ holes will be transferred to the AuNPs, forming a separation of ‘hot’ charges at the Au-TiO_2_ interface. However, owing to the close proximity of the charges at the interface, holes from the AuNPs may recombine quickly with the electrons in the conduction band of TiO_2_. This charge recombination process will lead to less H_2_ generated, resulting in a lower faradaic efficiency observed for the Au-TiO_2_ hollow spheres photo-anode/DSSN+-incorporated *E. coli* hybrid system.^41^

The working mechanism in the modified PEC–MFC hybrid system is proposed (Supplementary Figure S4). Upon illumination by a light source, electron–hole pairs are generated in the TiO_2_ photo-anode at the PEC cell.^42,43^ The introduction of the hollow sphere morphology allows for a higher probability of photon absorption, which directly increases the number of photo-generated electrons. The presence of metallic AuNPs facilitates charge separation and movement by minimising back flow of the photo-generated electrons (Supplementary Figure S4a). By using AuNPs, the visible light photoabsorption capability is also accentuated at ~580 nm wavelength. Green dots represent photo-generated electrons liberated during PEC water splitting at the Au-TiO_2_ hollow sphere photo-anode. These electrons then move through an external circuit to the MFC cathode, where they reduce the dissolved oxygen to water at the aerobic MFC cathode (Supplementary Figure S4b). In the MFC anode chamber, exogenously added COEs (DSSN+ and DSTN+) interact extensively with the cellular membrane of *E. coli*. This is attributed to their amphiphilic nature, which is realized by the hydrophobic portion of the delocalised π-conjugated aromatic backbone and long hydrophilic side chains.^24^ In addition, the pendant ionic end groups aid in solubility in aqueous media. The COEs intercalate and self-assemble in the cellular membrane of *E. coli* and facilitate enhanced extracellular charge transfer (Supplementary Figure S4c) via either aforementioned mechanism. The blue dots represent electrons liberated by *E. coli* during metabolism of the organic source and interaction with COEs. These electrons move towards the platinum counter electrode in the PEC cell to reduce protons (generated from PEC water splitting at the photo-anode) to form hydrogen gas. On the basis of collective data, successful incorporation of solar energy with oxidative breakdown of organic substrates by microbial catalytic mechanisms was demonstrated to achieve PEC water splitting at zero bias.

### Conclusions

In conclusion, by interfacing two seemingly independent platforms, the combined PEC–MFC hybrid system demonstrated significant improvement in photocurrent and hydrogen generation as compared with the independent PEC cell. The data presented herein points towards the novel application of COE-modified biofilm, with DSSN+ and DSTN+, and a unique photo-anode composition/morphology in Au-TiO_2_ for enhanced microbial electrohydrogenesis in a modified PEC–MFC hybrid system. The successful demonstration using these dual strategies provides exciting insights and paves the way forward for new discoveries towards a sustainable hydrogen economy.

## References

[bib1] Xi L , Chiam SY , Mak WF , Tran PD , Barber J , Loo SCJ et al. A novel strategy for surface treatment on hematite photoanode for efficient water oxidation. Chem Sci 2013; 4: 164–169.

[bib2] Tran PD , Nguyen M , Pramana SS , Bhattacharjee A , Chiam SY , Fize J et al. Copper molybdenum sulfide: a new efficient electrocatalyst for hydrogen production from water. Energ Environ Sci 2012; 5: 8912–8916.

[bib3] Tran PD , Pramana SS , Kale VS , Nguyen M , Chiam SY , Batabyal SK et al. Novel assembly of an MoS_2_ electrocatalyst onto a silicon nanowire array electrode to construct a photocathode composed of elements abundant on the earth for hydrogen generation. Chem Eur J 2012; 18: 13994–13999. 23008230 10.1002/chem.201202214

[bib4] Xi L , Tran PD , Chiam SY , Bassi PS , Mak WF , Mulmudi HK et al. Co_3_O_4_-decorated hematite nanorods as an effective photoanode for solar water oxidation. J Phys Chem C 2012; 116: 13884–13889.

[bib5] Ngaw CK , Xu Q , Tan TTY , Hu P , Cao S , Loo JSC . A strategy for *in situ* synthesis of well-defined core-shell Au@TiO_2_ hollow spheres for enhanced photocatalytic hydrogen evolution. Chem Eng J 2014; 257: 112–121.

[bib6] Xi L , Bassi PS , Chiam SY , Mak WF , Tran PD , Barber J et al. Surface treatment of hematite photoanodes with zinc acetate for water oxidation. Nanoscale 2012; 4: 4430–4433. 22688799 10.1039/c2nr30862b

[bib7] Gurudayal , Chiam SY , Kumar MH , Bassi PS , Seng HL , Barber J et al. Improving the efficiency of hematite nanorods for photoelectrochemical water splitting by doping with manganese. ACS Appl Mater Interfaces 2014; 6: 5852–5859. 24702963 10.1021/am500643y

[bib8] Hu P , Pramana SS , Cao S , Ngaw CK , Lin J , Loo SCJ et al. Ion-induced synthesis of uniform single-crystalline sulphide-based quaternary-alloy hexagonal nanorings for highly efficient photocatalytic hydrogen evolution. Adv Mater 2013; 25: 2567–2572. 23447427 10.1002/adma.201204545

[bib9] Cao S-W , Fang J , Shahjamali MM , Boey FYC , Barber J , Loo SCJ et al. Plasmon-enhanced hydrogen evolution on Au-InVO_4_ hybrid microspheres. RSC Adv 2012; 2: 5513–5515.

[bib10] Fang J , Cao S-W , Wang Z , Shahjamali MM , Loo SCJ , Barber J et al. Mesoporous plasmonic Au-TiO2 nanocomposites for efficient visible-light-driven photocatalytic water reduction. Int J Hydrogen Energ 2012; 37: 17853–17861.

[bib11] Long J , Chang H , Gu Q , Xu J , Fan L , Wang S et al. Gold-plasmon enhanced solar-to-hydrogen conversion on the {001} facets of anatase TiO_2_ nanosheets. Energ Environ Sci 2014; 7: 973–977.

[bib12] Subramanian V , Wolf EE , Kamat PV . Catalysis with TiO_2_/gold nanocomposites. effect of metal particle size on the Fermi level equilibration. J Am Chem Soc 2004; 126: 4943–4950. 15080700 10.1021/ja0315199

[bib13] Logan BE . Exoelectrogenic bacteria that power microbial fuel cells. Nat Rev Microbiol 2009; 7: 375–381. 19330018 10.1038/nrmicro2113

[bib14] Logan BE , Hamelers B , Rozendal R , Schröder U , Keller J , Freguia S et al. Microbial fuel cells: methodology and technology. Environ Sci Technol 2006; 40: 5181–5192. 16999087 10.1021/es0605016

[bib15] Wang VB , Chua S-L , Cao B , Seviour T , Nesatyy VJ , Marsili E et al. Engineering PQS biosynthesis pathway for enhancement of bioelectricity production in *Pseudomonas aeruginosa* microbial fuel cells. PLoS ONE 2013; 8: e63129. 23700414 10.1371/journal.pone.0063129 PMC3659106

[bib16] Wang VB , Du J , Chen X , Thomas AW , Kirchhofer ND , Garner LE et al. Improving charge collection in *Escherichia coli*-carbon electrode devices with conjugated oligoelectrolytes. Phys Chem Chem Phys 2013; 15: 5867–5872. 23487035 10.1039/c3cp50437a

[bib17] Wang VB , Yantara N , Koh TM , Kjelleberg S , Zhang Q , Bazan GC et al. Uncovering alternate charge transfer mechanisms in *Escherichia coli* chemically functionalized with conjugated oligoelectrolytes. Chem Commun 2014; 50: 8223–8226. 10.1039/c4cc02784a 24931387

[bib18] Hou H , Chen X , Thomas AW , Catania C , Kirchhofer ND , Garner LE et al. Conjugated oligoelectrolytes increase power generation in *E. coli* microbial fuel cells. Adv Mater 2013; 25: 1593–1597. 23345125 10.1002/adma.201204271

[bib19] Garner LE , Thomas AW , Sumner JJ , Harvey SP , Bazan GC . Conjugated oligoelectrolytes increase current response and organic contaminant removal in wastewater microbial fuel cells. Energ Environ Sci 2012; 5: 9449–9452.

[bib20] Wang H , Qian F , Wang G , Jiao Y , He Z , Li Y . Self-biased solar-microbial device for sustainable hydrogen generation. ACS Nano 2013; 7: 8728–8735. 24025029 10.1021/nn403082m

[bib21] Ito S , Chen P , Comte P , Nazeeruddin MK , Liska P , Péchy P et al. Fabrication of screen-printing pastes from TiO_2_ powders for dye-sensitised solar cells. Prog Photovoltaics Res Appl 2007; 15: 603–612.

[bib22] Wang P , Zakeeruddin SM , Comte P , Charvet R , Humphry-Baker R , Grätzel M . Enhance the performance of dye-sensitized solar cells by co-grafting amphiphilic sensitizer and hexadecylmalonic acid on TiO_2_ nanocrystals. J Phys Chem B 2003; 107: 14336–14341.

[bib23] Ito S , Murakami TN , Comte P , Liska P , Grätzel C , Nazeeruddin MK et al. Fabrication of thin film dye sensitized solar cells with solar to electric power conversion efficiency over 10%. Thin Solid Films 2008; 516: 4613–4619.

[bib24] Garner LE , Park J , Dyar SM , Chworos A , Sumner JJ , Bazan GC . Modification of the optoelectronic properties of membranes via insertion of amphiphilic phenylenevinylene oligoelectrolytes. J Am Chem Soc 2010; 132: 10042–10052. 20608655 10.1021/ja1016156

[bib25] Wang VB , Chua S-L , Cai Z , Sivakumar K , Zhang Q , Kjelleberg S et al. A stable synergistic microbial consortium for simultaneous azo dye removal and bioelectricity generation. Bioresour Technol 2014; 155: 71–76. 24434696 10.1016/j.biortech.2013.12.078

[bib26] Zhu C , Guo S , Wang P , Xing L , Fang Y , Zhai Y et al. One-pot, water-phase approach to high-quality graphene/TiO_2_ composite nanosheets. Chem Commun 2010; 46: 7148–7150. 10.1039/c0cc01459a 20657904

[bib27] Hoang S , Guo S , Hahn NT , Bard AJ , Mullins CB . Visible light driven photoelectrochemical water oxidation on nitrogen-modified TiO_2_ nanowires. Nano Lett 2011; 12: 26–32. 22112010 10.1021/nl2028188

[bib28] He Z , Guai G , Liu J , Guo C , Chye Loo JS , Li CM et al. Nanostructure control of graphene-composited TiO_2_ by a one-step solvothermal approach for high performance dye-sensitized solar cells. Nanoscale 2011; 3: 4613–4616. 22006266 10.1039/c1nr11300c

[bib29] Zhang Z , Wang Z , Cao S-W , Xue C . Au/Pt nanoparticle-decorated TiO_2_ nanofibers with plasmon-enhanced photocatalytic activities for solar-to-fuel conversion. J Phys Chem C 2013; 117: 25939–25947.

[bib30] Chen M , Hu L , Xu J , Liao M , Wu L , Fang X . ZnO hollow-sphere nanofilm-based high-performance and low-cost photodetector. Small 2011; 7: 2449–2453. 21780283 10.1002/smll.201100694

[bib31] Dong K , Liu Z , Ren J . A general and eco-friendly self-etching route to prepare highly active and stable Au@metal silicate yolk-shell nanoreactors for catalytic reduction of 4-nitrophenol. CrystEngComm 2013; 15: 6329–6334.

[bib32] Dong Z , Lai X , Halpert JE , Yang N , Yi L , Zhai J et al. Accurate control of multishelled ZnO hollow microspheres for dye-sensitized solar cells with high efficiency. Adv Mater 2012; 24: 1046–1049. 22266874 10.1002/adma.201104626

[bib33] Wu J-L , Chen F-C , Hsiao Y-S , Chien F-C , Chen P , Kuo C-H et al. Surface plasmonic effects of metallic nanoparticles on the performance of polymer bulk heterojunction solar cells. ACS Nano 2011; 5: 959–967. 21229960 10.1021/nn102295p

[bib34] Baek S-W , Park G , Noh J , Cho C , Lee C-H , Seo M-K et al. Au@Ag core-shell nanocubes for efficient plasmonic light scattering effect in low bandgap organic solar cells. ACS Nano 2014; 8: 3302–3312. 24593128 10.1021/nn500222q

[bib35] Hirakawa T , Kamat PV . Charge separation and catalytic activity of Ag@TiO_2_ core-shell composite clusters under UV-irradiation. J Am Chem Soc 2005; 127: 3928–3934. 15771529 10.1021/ja042925a

[bib36] Li Y , Wang H , Feng Q , Zhou G , Wang Z-S . Gold nanoparticles inlaid TiO_2_ photoanodes: a superior candidate for high-efficiency dye-sensitized solar cells. Energ Environ Sci 2013; 6: 2156–2165.

[bib37] Sivakumar K , Wang V , Chen X , Bazan G , Kjelleberg S , Loo S et al. Membrane permeabilization underlies the enhancement of extracellular bioactivity in *Shewanella oneidensis* by a membrane-spanning conjugated oligoelectrolyte. Appl Microbiol Biotechnol 2014, 98: 9021–9031. 25091046 10.1007/s00253-014-5973-3

[bib38] Wang VB , Kirchhofer ND , Chen X , Tan MYL , Sivakumar K , Cao B et al. Comparison of flavins and a conjugated oligoelectrolyte in stimulating extracellular electron transport from *Shewanella oneidensis* MR-1. Electrochem Commun 2014; 41: 55–58.

[bib39] Yu Y , Cao CY , Chen Z , Liu H , Li P , Dou ZF et al. Au nanoparticles embedded into the inner wall of TiO2 hollow spheres as a nanoreactor with superb thermal stability. Chem Commun 2013; 49: 3116–3118. 10.1039/c3cc39212k 23467689

[bib40] Hisatomi T , Kubota J , Domen K . Recent advances in semiconductors for photocatalytic and photoelectrochemical water splitting. Chem Soc Rev 2014; 43: 7520–7535. 24413305 10.1039/c3cs60378d

[bib41] Brennan L , Purcell-Milton F , Salmeron A , Zhang H , Govorov A , Fedorov A et al. Hot plasmonic electrons for generation of enhanced photocurrent in gold-TiO_2_ nanocomposites. Nanoscale Res Lett 2015; 10: 38. 25852335 10.1186/s11671-014-0710-5 PMC4385105

[bib42] Wang G , Wang H , Ling Y , Tang Y , Yang X , Fitzmorris RC et al. Hydrogen-treated TiO_2_ nanowire arrays for photoelectrochemical water splitting. Nano Lett 2011; 11: 3026–3033. 21710974 10.1021/nl201766h

[bib43] Feng X , Shankar K , Varghese OK , Paulose M , Latempa TJ , Grimes CA . Vertically aligned single crystal TiO2 nanowire arrays grown directly on transparent conducting oxide coated glass: synthesis details and applications. Nano Lett 2008; 8: 3781–3786. 18954124 10.1021/nl802096a

