# Supplementary material for: Enhancement in hydrogen evolution using Au-TiO2 hollow spheres with microbial devices modified with conjugated oligoelectrolytes
Source: NPJ Biofilms Microbiomes. 2015 Oct 21;1:15020–. doi: 10.1038/npjbiofilms.2015.20 (PMC5515218; doi:10.1038/npjbiofilms.2015.20)
Supplement: Supplementary Information [file npjbiofilms201520-s1.doc]

**Enhancement in hydrogen evolution using Au-TiO_2_ hollow spheres with microbial devices modified with conjugated oligoelectrolytes**

Chee Keong Ngaw^1, 2, 3|^, Victor Bochuan Wang^4, 5|^, Zhengyi Liu^2^, Yi Zhou^4^, Staffan Kjelleberg^5, 6^, Qichun Zhang^4*^, Timothy Thatt Yang Tan^2*^, Say Chye Joachim Loo^3, 4, 5*^

1. Energy Research Institute @ NTU (ERI@N), Interdisciplinary Graduate School, Nanyang Technological University, 50 Nanyang Avenue, Singapore 639798, Singapore
2. Solar Fuels Laboratory, School of Chemical and Biomedical Engineering, Nanyang Technological University, 62 Nanyang Drive, Singapore 627459, Singapore
3. Solar Fuels Laboratory, School of Materials Science and Engineering, Nanyang Technological University, 50 Nanyang Avenue, Singapore 639798, Singapore
4. School of Materials Science and Engineering, Nanyang Technological University, Singapore 639798, Singapore
5. Singapore Centre on Environmental Life Sciences Engineering (SCELSE), Nanyang Technological University, Singapore 637551, Singapore
6. School of Biotechnology and Biomolecular Sciences and Centre for Marine Bio-Innovation, The University of New South Wales, Sydney NSW 2052, Australia

| Equal contribution

*Corresponding author

**Supplementary Information**

**
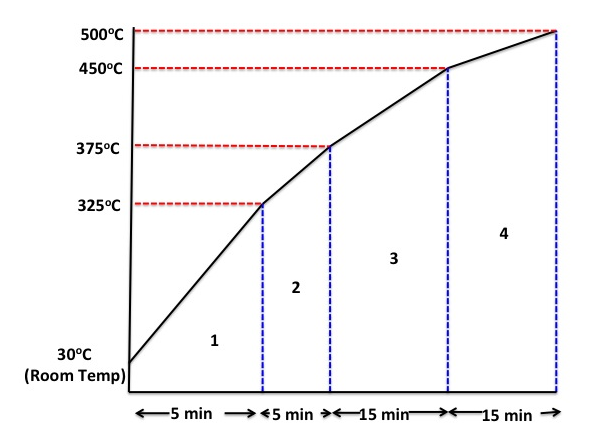
**

**Figure S1.** Heating cycle for synthesizing electrode. The coated FTO substrate was gradually heated in the muffle furnace at 325 ^o^C/5 min, 375 ^o^C/5 min, 450 ^o^C/15 min, 500 ^o^C/15 min to obtain the electrode.

**
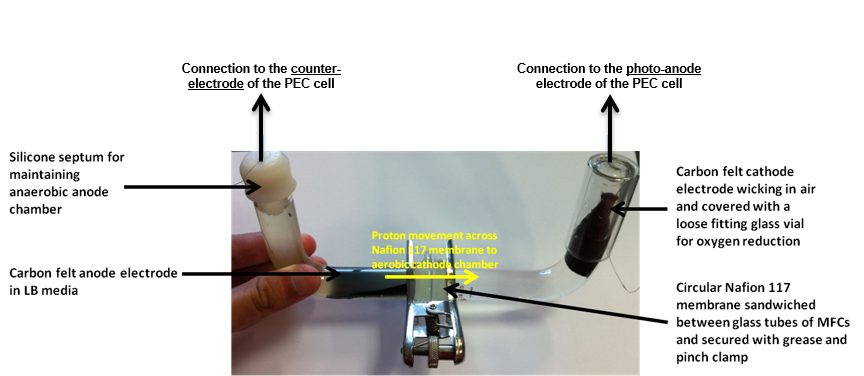
**

**Figure S2.** Photo of the MFC used in the PEC-MFC hybrid system.







**Figure S3.** (a) Linear sweep voltammograms and (b) Amperometric current-time curves of hybrid PEC-MFC system employing different photo-anodes and *E. coli* chemically modified with DSTN+ and DSSN+. The electrolyte is 0.5 M Na_2_SO_4_ and the scan rate is 20 mV/s. Poising potential is 0 V vs. Pt, with light on-off cycle at light intensity of 100 mW/cm^2^.

**
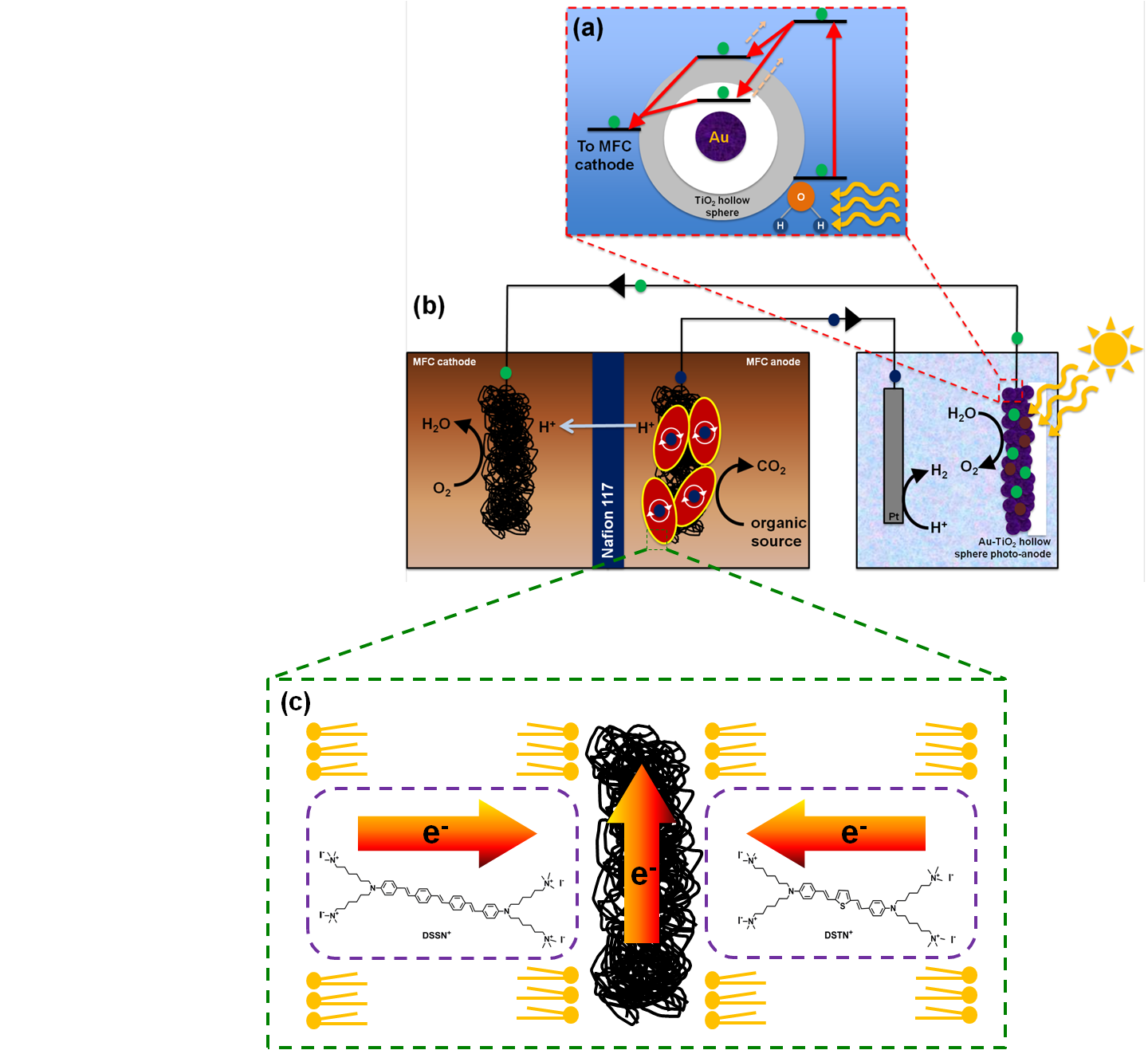
**

**Figure S4.** Working mechanisms in PEC-MFC hybrid system with chemically modified photo-anode and *E. coli*. (a) Schematic representation of charge separation and movement in Au-TiO_2_ hollow sphere photo-anode. (b) Mechanistic diagram of the PEC-MFC hybrid system. Green dots represent electrons liberated during photoelectrochemical water splitting at the Au-TiO_2_ hollow sphere photo-anode. Blue dots represent electrons liberated by *E. coli* during metabolism of the organic source and interaction with COEs. (c) Schematic representation of DSSN+ and DSTN+ incorporation in lipid membranes of *E. coli*. Chemical structures of DSSN+ and DSTN+ are shown in hatched boxes.

**Synthesis procedures and characterization of DSTN+**

**
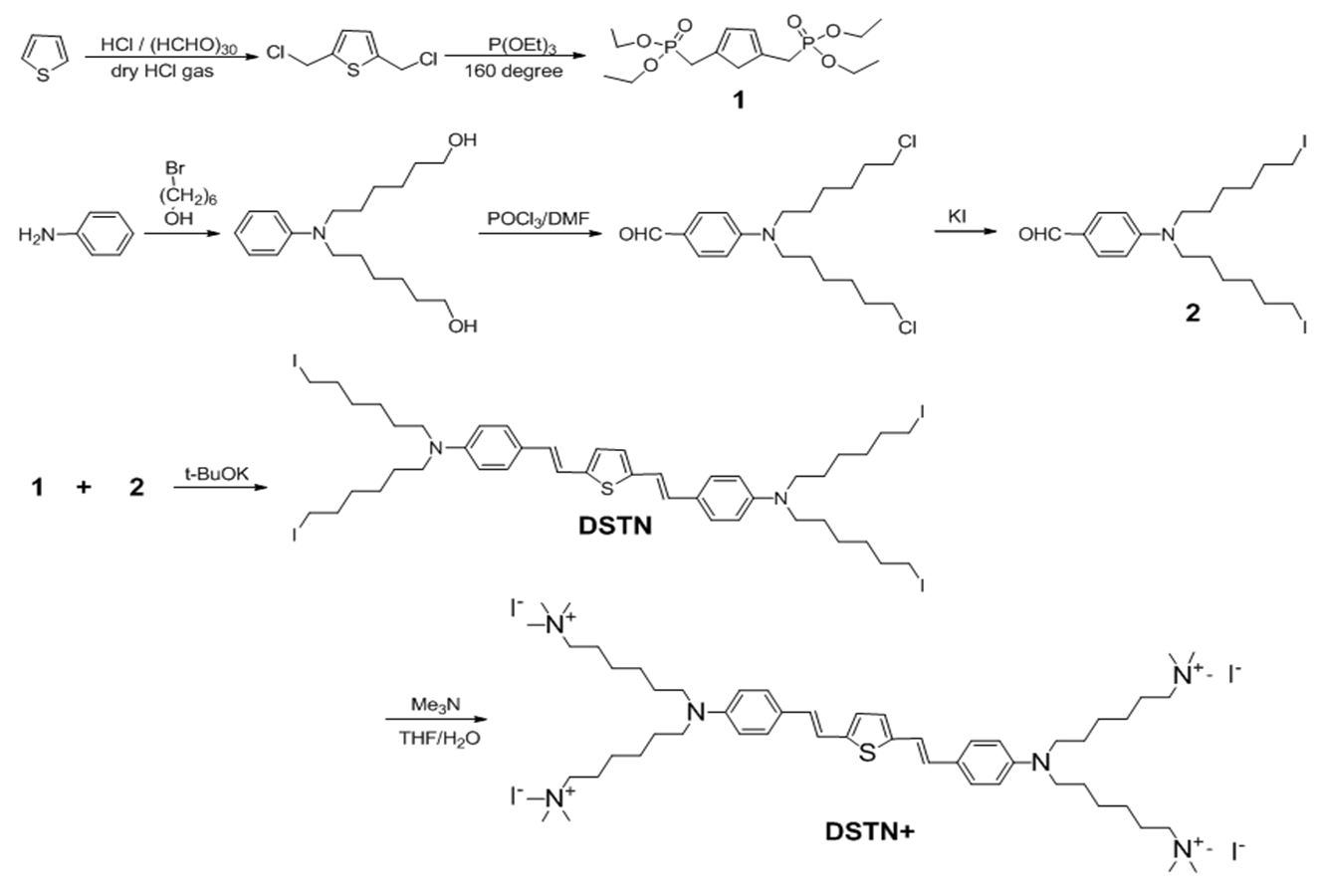
**

**Tetraethyl-(cyclopenta-3,5-diene-1,3 diylbis(methylene))bis(phosphonate) (1):** Thiophene (50 mmol, 4.2 g), paraformaldehyde (58.5 mmol, 1.75 g), and concentrated hydrogen chloride (6.4 mL) were added to a 100 mL three-necked flask at -5°C to 0°C. Gaseous dry hydrogen chloride was added slowly under stirring over a period of 2 h. After further reaction for 2 h, the resulting mixture was poured into distilled water. The organic phase was collected and the excess thiophene was removed with rotary evaporation. The obtained brown oil was reacted with 10 mL triethyl phosphite in a 100 mL flask at 160°C for 3 h. Excess triethyl phosphite was removed by vacuum distillation. Compound **1** was obtained as a dark yellow oil (9.7 g, yield: 53%) by column chromatography (SiO_2_, hexane/EA 4:1).^1^H NMR (400 MHz, CDCl_3_): δ = 6.71 (s, 2H, Ar-H), 3.96 (dd, *J* = 13.4, 6.5 Hz, 8H, -CH_2_-), 3.19 (d, *J* = 19.5 Hz, 4H, -CH_2_-), 1.18 (t, *J* = 6.9 Hz, 12H, -CH_3_).

**
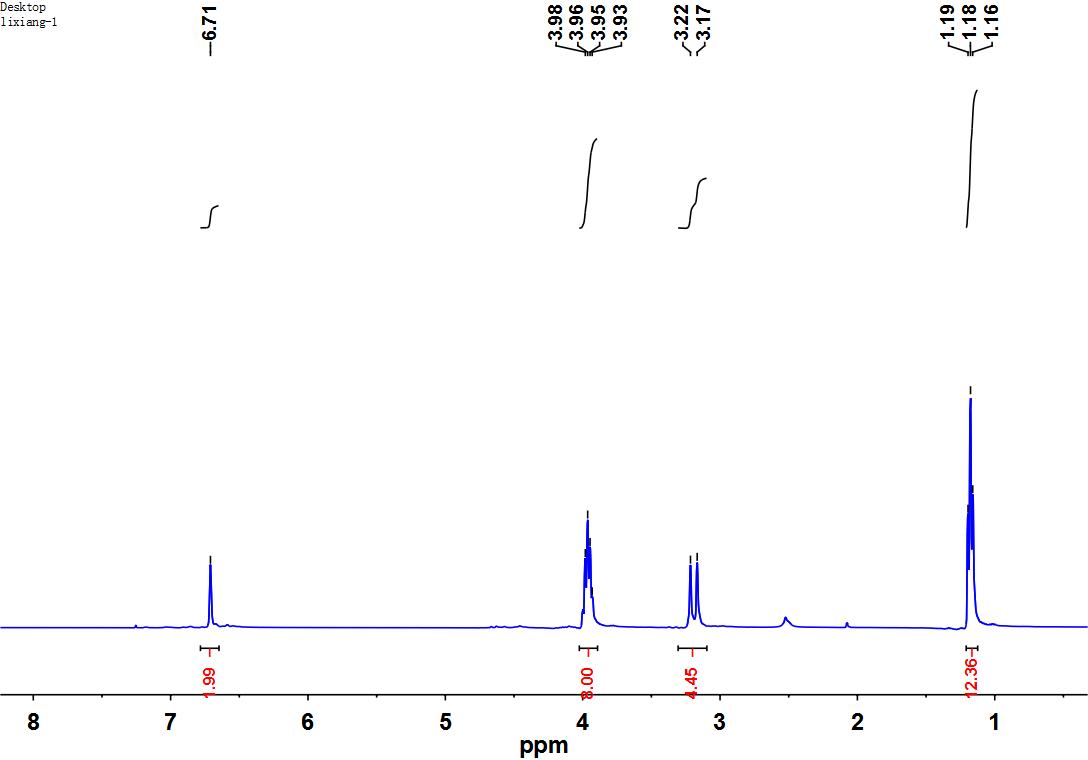
**

**N,N-Bis(6'-iodohexyl)-4-aminobenzaldehyde (2):** Compound 2 was synthesized according to a reported literature[^1^](#_ENREF_1).

**4,4'-thiophene-2,5-diylbis(ethene-2,1-diyl))bis(N,N-bis(6-iodohexyl)aniline) (DSTN):** To a solution containing **1** (0.5 g, 1.3 mmol) and aldehyde **2** (1.76 g, 3.25 mmol) in dry THF (50 mL), potassiumtert-butoxide (3.6 mmol, 0.4 g) was added in portions. The resulting suspension was stirred overnight at room temperature, poured into 200 mL distilled water, extracted with dichloromethane, and washed with brine for three times. The organic layer was dried over MgSO_4_. The solvent was evaporated and the residue was further purified by column chromatography (SiO_2_: hexane/DCM 1:1) to give DSTN as a brown oil (1.04 g, yield: 70 %). ^1^H NMR (400 MHz, CDCl_3_): δ = 7.32 (d, *J* = 8.2 Hz, 4H, Ar-H), 6.96 (d, *J* = 15.9 Hz, 2H, Ar-H), 6.80 (d, *J* = 17.1 Hz, 4H, Ar-H), 6.60 (d, *J* = 8.3 Hz, 4H, Ar-H), 3.55 (t, *J* = 6.6 Hz, 3H, -CH_2_-), 3.28 (s, 8H, -CH_2_-), 3.20 (t, *J* = 6.9 Hz, 5H, -CH_2_-), 1.95-1.73 (m, 2H, -CH_2_-), 1.67-1.58 (m, 2H, -CH_2_-), 1.46 (dt, *J* = 14.8, 7.7 Hz, 2H, -CH_2_-), 1.36 (d, *J* = 4.9 Hz, 2H, -CH_2_-).


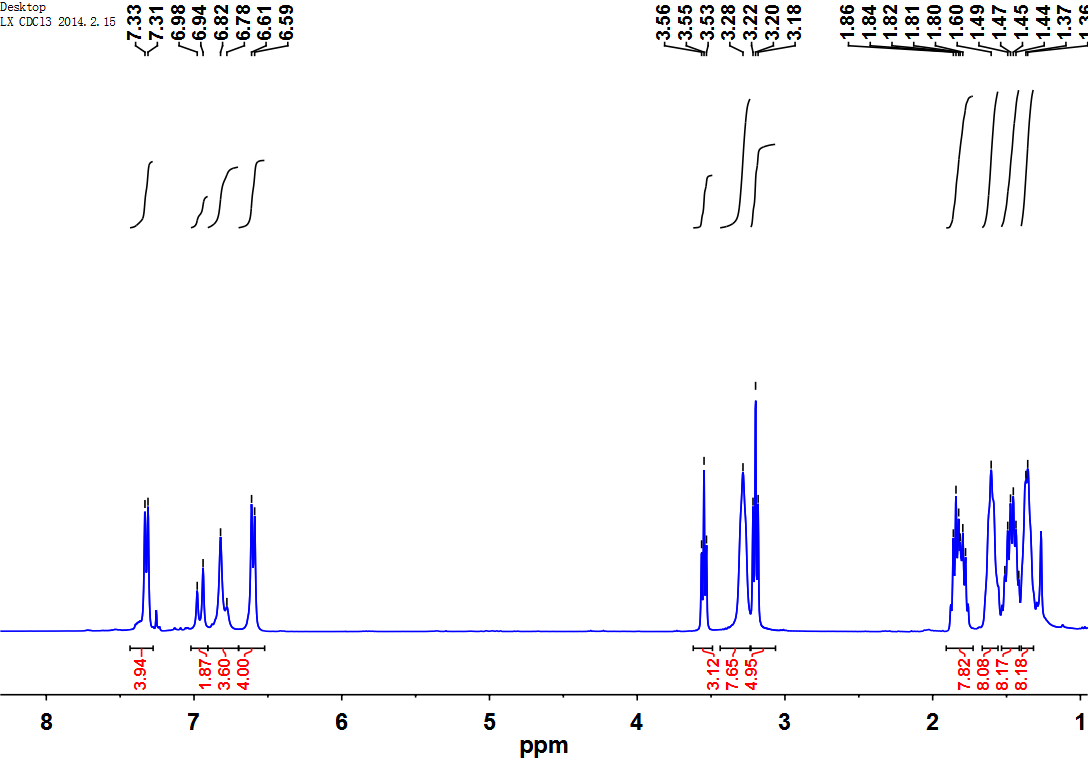


**6,6',6'',6'''-(((-thiophene-2,5-diylbis(ethene-2,1-diyl))bis(4,1-phenylene))bis(azanetriyl))tetrakis(N,N,N-trimethylhexan-1-aminium) iodide (DSTN+):** To a solution of **DSTN** (400 mg, 0.35 mmol) in anhydrous THF (20 mL) under argon atmosphere, excess amount of NMe_3_ (in ethanol 33%) was injected to the flask with a syringe. The reaction solution was heated to 60°C for 24 h. When the reaction was completed, the solvent and excess NMe_3_ were removed via rotary evaporation. The product was precipitated with ether and washed three times to give DSTN+ as a yellow solid (320 mg, yield: 67 %). ^1^H NMR (400 MHz, CDCl_3_): δ = ^1^H NMR (400 MHz, DMSO) δ 7.32 (d, *J* = 8.1 Hz, 4H, Ar-H), 7.04 (d, *J* = 16.0 Hz, 2H, Ar-H), 6.92 (s, 2H, Ar-H), 6.70 (d, *J* = 15.9 Hz, 2H, Ar-H), 6.61 (d, *J* = 8.0 Hz, 4H, Ar-H), 3.28 (m, 16H, -CH_2_-), 3.02 (s, 36H, -CH_3_), 1.65 (m, 8H, -CH_2_-), 1.52 (m, 8H, -CH_2_-), 1.31 (m, 16H, -CH_2_-). *ESI*-MS: m/z 1395.4 [M+H]^+^.


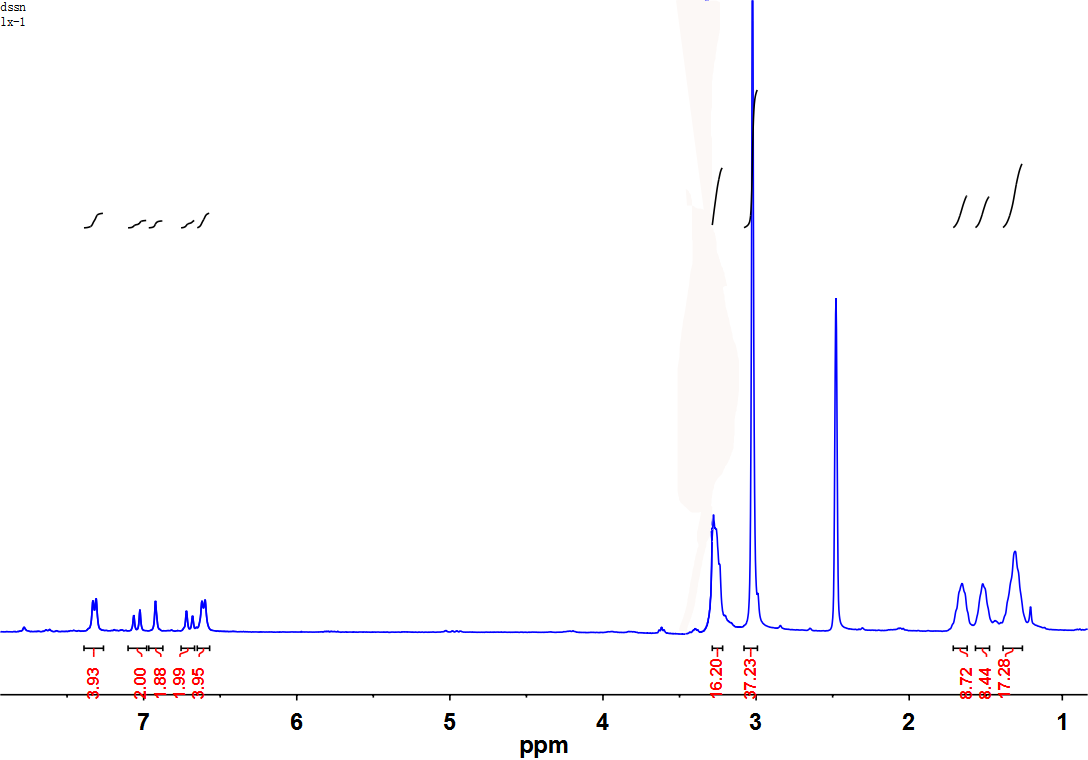


**References**

1. H. Y. Woo, B. Liu, B. Kohler, D. Korystov, A. Mikhailovsky and G. C. Bazan, *Journal of the American Chemical Society*, 2005, **127**, 14721-14729.
